# Roles of Anatomical Abnormalities in Localized and Diffuse Chronic Rhinosinusitis

**DOI:** 10.1007/s12070-022-03370-0

**Published:** 2023-02-10

**Authors:** Lei Liu, Qian Chen, Min Pan, Yucheng Yang

**Affiliations:** 1 Department of Otorhinolaryngology, The First Affiliated Hospital of Chongqing Medical University, Chongqing, China; 2 Department of Otolaryngology, The First Affiliated Hospital, Chongqing Medical University, No.1 Youyi Road, Yuzhong District, 400016 Chongqing, China

**Keywords:** Sinus, Anatomic Variants, Chronic Rhinosinusitis, Computed Tomography

## Abstract

**Background:**

This study aimed to examine the roles of anatomical variation in localized and diffuse chronic rhinosinusitis [LCRS and DCRS]) .

**Methods:**

A database was analyzed retrospectively on patients hospitalized in the Department of Otorhinolaryngology of our university hospital from 2017 to 2020. A total of 281 patients were included and divided into three categories: patients with LCRS, patients with DCRS, and a normal control group. The frequency of anatomical variation, the demographic information, disease type (with or without polyps), symptom visual analogue scale (VAS) scores and Lund-Mackay (L-M) scores were calculated and compared.

**Results:**

Anatomical variants were observed more frequently in LCRS than DCRS (P < 0.05). The frequency of variation was higher in the LCRSwNP group than the DCRSwNP group (P < 0.05), and higher in the LCRSsNP group than the DCRSsNP group (P < 0.05). The L-M scores for patients with DCRS with nasal polyps were significantly higher (14.96 ± 6.15) than those of patients with DCRS without nasal polyps (6.80 ± 5.00) and also significantly higher (3.78 ± 2.07) than those of patients with LCRS with nasal polyps (2.63 ± 1.12; P < 0.05). A poor correlation was observed between the severity of symptoms and the performance of CT scans in CRS (R = 0.29, P < 0.01).

**Conclusion:**

Anatomical variants were common in CRS, and possibly correlated with LCRS but not with DCRS. The frequency of anatomical variation is not associated with the occurrence of polyps. CT could reflect the severity of disease symptoms to some extent.

## Introduction

Chronic sinusitis (CRS) is defined by the European Position Paper on Rhinosinusitis and Nasal Polyps 2020 (EPOS2020) as a chronic inflammatory disease of the paranasal sinuses that lasts for more than 12 weeks and includes at least one of the two symptoms of nasal obstruction and purulent nose, and at least one other symptom, such as headache, and reduction or loss of smell [1]. CRS occurs in > 10% of the adult population in Europe and the.

USA [2]. Annual incremental costs were $11,507 higher for patients with CRSwNP versus those without CRS [3]. Patients with CRS may also have other inflammatory airway conditions such as asthma and allergic rhinitis.

The guidelines propose a new CRS classification into primary and secondary CRS, which are further divided into localized (unilateral) chronic rhinosinusitis (LCRS) and diffuse (bilateral) chronic rhinosinusitis (DCRS) [1]. Some retrospective study reported that the incidence of unilateral sinus lesions was 25% in all cases, the most common being chronic sinusitis [4, 5]. The pathogenesis of CRS is unclear and is considered multifactorial. Potential risk factors for CRS include genetic, anatomical, and environmental factors and comorbid diseases [2]. Anatomical variation exists among individuals, and its role in the pathogenesis of CRS remains unclear. Surgical treatment strategy of CRS is adjusted to correct anatomical abnormalities so as to drainage. It is therefore crucial to further investigate the anatomical variation.

A prior series of studies [6–9] have shown significant anatomical variation in chronic sinusitis. Jain et al. [10] suggested that the anatomical abnormalities in patients with diffuse sinusitis are similar to those in healthy controls, whereas significantly more anatomical abnormalities are found in patients with localized sinusitis than patients with diffuse sinusitis. In addition, the authors suggested that localized sinusitis is more likely to be associated with the anatomical variation in the ostium complex of the nasal meatus and the middle nasal meatus, whereas diffuse sinusitis is more likely to result from systemic mucosal abnormalities of the anatomically inconspicuous paranasal sinuses. However, Capelli et al. [11] reported that thickening of the maxillary mucosa greater than that of 2 mm and closure of the maxillary sinus orifice are firmly associated with CRS, whereas no significant correlation exists between common anatomical variations and CRS.

To date, research evidence regarding the significance of anatomical variation in the new CRS classification or the correlation between nasal polyps and anatomical variation is scarce. A review article has concluded that no evident correlation exists between CRS and nasal septum deviation [12]. Another review suggested that CRS may be associated with nasal septum deviation of more than 10°. Furthermore, that review supports the conclusion that in patients with CRS, nasal septum deviation does not contribute significantly to CRS symptom severity but may affect sinus mucosal hyperplasia [13]. Thus, Owing to the complexity of nasal septum variation precision measurement and unpredictable factors such as clinical symptoms, nasal septum deviation was not examined in this study. The anatomical abnormalities recorded in this study were: agger nasi cells, concha bullosa, uncinate process variations, paradoxical middle turbinate, Haller cells, accessory ostia and Onodi cells.

To figure out the severity of paranasal sinus disease and its correlation with the frequency of anatomical variation, we compared the anatomical variation in patients with limited and diffuse CRS to patients without sinus symptoms.

## Materials & Methods

### Study Participants

A total of 281 patients with CRS and healthy examiner (general public for health check-ups who suffered no nasal symptoms) were collected from January 2017 to January 2020. All patients with CRS were diagnosed with chronic sinusitis on the basis of medical history collection, physical examination by a specialist, imaging examination and other related auxiliary examinations such as nasal endoscopy. No statistical differences were found in sex and age between groups. Thus, the data for the two groups were comparable. The study was approved by the Institutional Review Board of The First Affiliated Hospital of Chongqing Medical University (No. 2021 − 107).

### Inclusion Criteria

1. CRS group met the diagnostic criteria of EPOS2020.
2. Control group were general public for health check-ups without nasal symptoms. (The VAS scores is 0)

### Exclusion Criteria

1. Age less than 14 years.
2. Lack of complete clinical data.
3. Previous history of paranasal sinus surgery.
4. Previous lesions of the nasal cavity and paranasal sinus such as nasal tumors, fungal sinusitis, allergic sinusitis and asthma.
5. Other systemic diseases such as immunodeficiency.

### Data Collection

Patients were selected according to the inclusion and exclusion criteria, and CRS was divided into an LCRS group and DCRS group according to the EPOS2020 classification criteria. According to the presence or absence of polyps, they was divided into the following groups: localized chronic sinusitis with polyps (LCRSwNP), localized chronic sinusitis without polyps (LCRSsNP), diffuse chronic sinusitis with polyps (DCRSwNP) and diffuse chronic sinusitis without polyps (DCRSsNP).

Thin slice computed tomography (CT) images of the coronal, sagittal and axial planes were reviewed. The collected data included agger nasi cells, concha bullosa, uncinate process variations, paradoxical middle turbinate, accessory ostia, Onodi cells and Haller cells.（Fig. 2） In addition, the L-M score and the symptoms VAS scores were calculated.

### Statistical Methods

SPSS22.0 statistical software was used for independent sample dichotomous data χ² tests, Mann-Whitney U tests and Kruskal-Wallis H tests of nonparametric rank sum tests, and statistically significant differences were determined.

## Results

### Patient Information

A total of 281 participants who met the inclusion criteria were included in this study. The cases comprised 43 cases in the LCRS group (27 in the LCRSwNP group and 16 in the LCRSsNP group) and 117 cases in the DCRS group (97 in the DCRSwNP group and 20 in the DCRSsNP group). The total number of control group was 121. As shown in Table 1, the LCRSwNP group included 13 cases (48.1%) in males and 14 cases (51.9%) in females, with a mean age of 41.63 ± 18.25 years, VAS score of 3.78 ± 2.07, Lund-Mackay score of CT 6.78 ± 3.92 and variation frequency (number of variation per patient) of 2.63 ± 1.12. The LCRSsNP group included nine cases in males (56.3%) and seven cases in females (43.7%), with a mean age of 46.00 ± 17.39 years, VAS score of 2.98 ± 1.95, Lund-Mackay score of CT 5.56 ± 3.05 and variation frequency of 3.31 ± 1.49. There were 63 (64.9%) males and 35 (35.1%) females in the DCRSwNP group, with a mean age of 45.59 ± 11.71 years, VAS score of 4.31 ± 1.80, Lund-Mackay score of CT 14.96 ± 6.15 and variation frequency of 1.59 ± 0.99. The DCRSsNP group included 12 (60.0%) males and 8 (40.0%) females, with a mean age of 46.95 ± 14.74 years, VAS score of 3.51 ± 1.59, Lund-Mackay score of CT 6.80 ± 5.00 and variation frequency of 2.00 ± 1.21. The control group included 47 (38.8%) males and 74 (61.2%) females, with a mean age of 45.72 ± 15.47 years and a variation frequency of 1.50 ± 0.96.

**Table 1:** General information, VAS, Lund-Mackay scores and frequency of variation for each subgroup of patients

	LCRS		DCRS		Control	*P* value
	wNP	sNP	wNP	sNP		
Gender						
Male	13(48.1%)	9(56.3%)	63(84.0%)	34(80.9%)	47(38.8%)	<0.001
Female	14(51.9%)	7(43.7%)	12(16.0%)	8(19.1%)	74(61.2%)	
Age(years)	41.63 ± 18.25	46.00 ± 17.39	45.59 ± 11.71	46.95 ± 14.74	45.72 ± 15.47	0.577
VAS scores	3.78 ± 2.07	2.98 ± 1.95	4.31 ± 1.80	3.51 ± 1.59	/	<0.001
Lund-Mackay scores	6.78 ± 3.92	5.56 ± 3.05	14.96 ± 6.15	6.80 ± 5.00	/	<0.001
frequency of variation	2.63 ± 1.12	3.31 ± 1.49	1.59 ± 0.99	2.00 ± 1.21	1.50 ± 0.96	<0.001

VAS = visual analogue scale, LCRS = localized chronic rhinosinusitis, DCRS = diffuse chronic rhinosinusitis, wNP = with nasal polyps, sNP = without nasal polyps, frequency of variation means the number of variation per patient, * means ± SD,

### Symptom VAS Scores

**Fig. 1:**
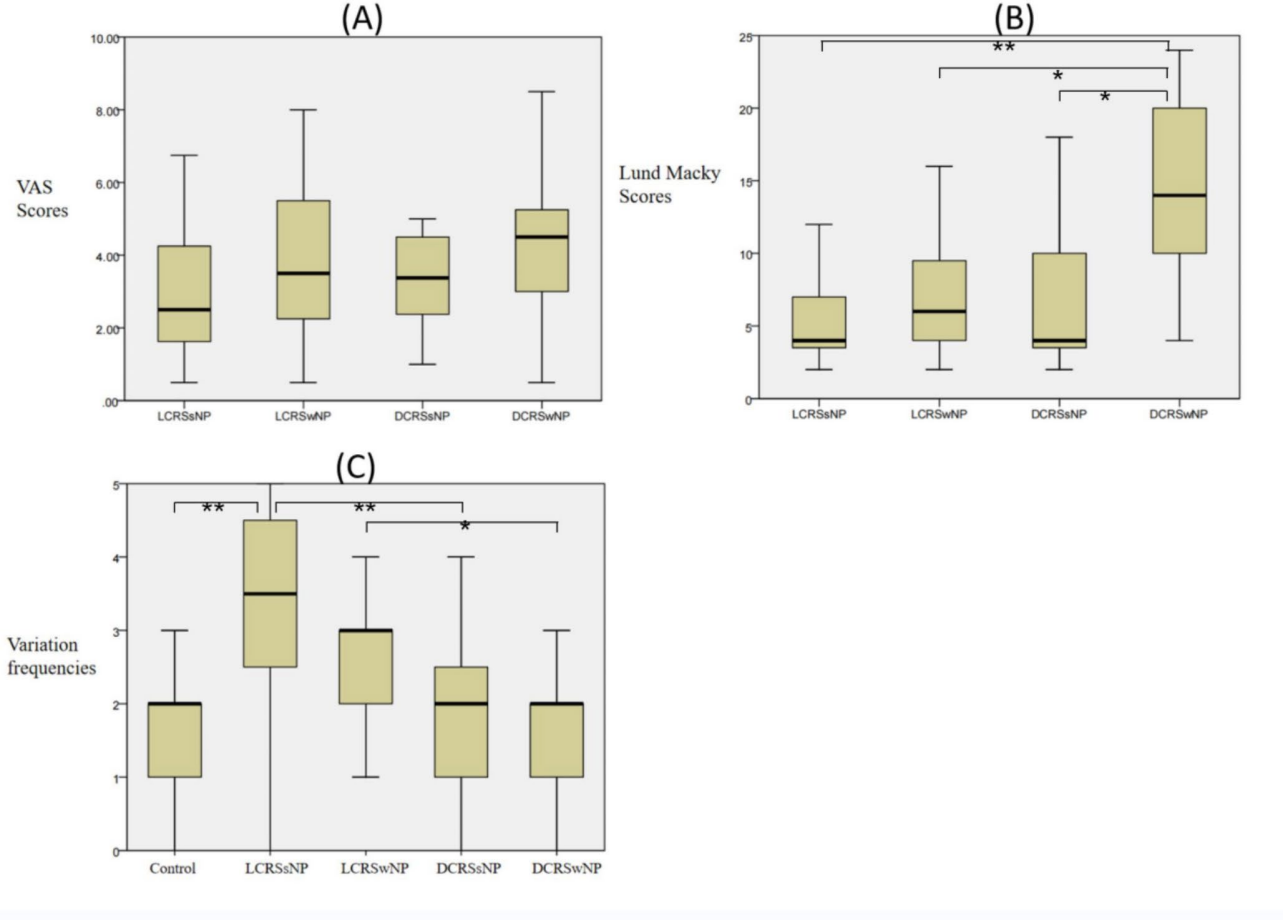
Box plot of for each subgroup of chronic rhinosinusitis:(A) VAS scores (B) Lund-Macky scores (C) Variation frequencies, * means P<0.05, ** means P<0.01

The VAS scores for overall symptoms were lower in the LCRS group (3.48 ± 2.04) than the DCRS group (4.17 ± 1.78) (P < 0.05), but no statistically significant difference was observed between any two subgroups (P > 0.05). The distribution of the overall symptom VAS score of each subgroup of CRS indicated that the symptoms of LCRS were milder than those of DCRS, but the presence or absence of polyps had no effect on the symptoms (Fig. 1).

In addition, as shown in Table 2, symptoms such as nasal obstruction, nasal discharge and headache did not significantly differ between the LCRS and DCRS groups (P > 0.05), but the symptoms of reduction of smell in the LCRS group were milder than those in the DCRS group (P < 0.05).

**Table 2:** VAS scores of localized and diffuse chronic sinusitis

	Obstruction	Discharge	Reduction of smell	Headache	Overall
LCRS	4.67	3.74	2.86	1.88	3.48
DCRS	5.56	3.5	5.26	2.64	4.17
Z	-1.600	-0.329	-3.482	-1.002	-1.979
*P*	0.110	0.742	0.000	0.316	0.048

VAS = visual analogue scale, LCRS = localized chronic rhinosinusitis, DCRS = diffuse chronic rhinosinusitis

### Lund-Mackay Scores

As shown in Table 3, the maxillary sinus was the most frequently involved sinus, and no significant difference was observed between the LCRS group and the DCRS group (P > 0.05). However, the involvement of the ethmoid sinus, sphenoid sinus and frontal sinus in the DCRS group was significantly higher than that in the LCRS group (P < 0.05). Therefore, we speculated that the pathogenesis of LCRS may be associated with stenosis of the maxillary sinus ostium and surrounding structures caused by anatomical variations.

**Table 3:** Involvement of sinuses in localized and diffuse chronic sinusitis

	Maxillary sinus	Ethmoid sinus	Sphenoid sinus	Frontal sinus
LCRS	41(95.3%)	28(65.1%)	14(32.5%)	13(30.2%)
DCRS	110(94.0%)	108(92.3%)	63(53.8%)	76(64.9%)
χ²	0.105	18.234	5.708	15.360
*P*	0.746	0.000	0.017	0.000

LCRS = localized chronic rhinosinusitis, DCRS = diffuse chronic rhinosinusitis

The distribution of CT scores in each subgroup of CRS is shown in Fig. 1. The L-M score was significantly higher for DCRSwNP than both DCRSsNP and LCRSwNP (P < 0.05). No significant statistical differences were observed between LCRSwNP and LCRSsNP (2.98 ± 1.95; P > 0.05). Thus, polyps do not reflect the severity observed in CT scans.

### Frequency of Anatomical Variation

The frequency of anatomical variation in each subgroup is shown in Table 4. There was no significant difference between CRS group and control group for the prevalence of agger nasi cells and Haller cells (P > 0.05). The incidence of concha bullosa, uncinate process variations, paradoxical middle turbinate, accessory ostia and Onodi cells in the CRS groups was higher than that in the control group (P < 0.05).

**Table 4:** Different anatomical variants in each subgroup

	AC	CB	PMT	UPV	AO	OC	HC
LCRS							
wNP	13(48.1%)^*^	8(29.6%)	5(18.5%)	20(74.1%)	8(29.6%)	15(55.6%)	3(11.1%)
sNP	13(81.3%)	6(37.5%)	7(43.8%)	10(62.5%)	10(62.5%)	10(62.5%)	3(18.8%)
DCRS							
wNP	56(57.1%)	10(10.0%)	6(6.2%)	17(85.0%)	19(19.6%)	35(36.1%)	12(12.4%)
sNP	17(85.0%)	4(20.0%)	3(15.0%)	48(49.5%)	11(55.0%)	14(70.0%)	2(10.0%)
Control	74(61.2%)	15(12.4%)	3(2.5%)	46(38.0%)	3(2.5%)	22(18.2%)	16(13.2%)

AC = Agger nasi cell, CB = Concha bullosa, PMT = Paradoxical middle turbinate, UPV = Uncinate process variations, AO = Accessory ostia, OC = Onodi cells, HC = Haller cells, * =n(n%), LCRS = localized chronic rhinosinusitis, DCRS = diffuse chronic rhinosinusitis, wNP = with nasal polyps, sNP = without nasal polyps

The distribution of anatomical variation in each subgroup is shown in Fig. 1. The control group showed lower frequency of variation than the LCPSwNP and LCRSsNP groups (P < 0.05), but showed no statistically significant difference compared to DCRSwNP and DCRSsNP (P > 0.05). Simultaneously, we observed no statistical difference in the frequency of anatomical variation with or without polyps in the subgroups of LCRS and DCRS (P > 0.05). It should be noted that the frequency of variation was higher in the LCRSwNP group than the DCRSwNP group (P < 0.05), and higher in the LCRSsNP group than the DCRSsNP group (P < 0.05). The anatomical variation in LCRS was significantly more frequent than that in DCRS, and the difference between DCRS and normal condition was not statistically significant, thus, indicating that anatomical variation may be a cause of LCRS.

### Correlation Analysis

As shown in Table 5, we further statistically analyzed the correlations among the factors we noticed and found that: (1) as the age of onset increased, the frequency of anatomical variations in patients gradually decreased (P < 0.05) ; (2) patients’ evaluations of their own symptoms were worse in females than males (P < 0.05); (3) when CRS was bilateral with polyps, patient symptoms and CT manifestations were more severe (P < 0.01); and (4) when the disease was unilateral, anatomical variation was more frequent, and the L-M score on CT scans was lower (P < 0.01).

**Table 5:** Correlation analysis of multiple statistical factors

	Sex	Age	VAS scores	Lund Macky scores	Frequency of variation	Unilateral or bilateral	Nasal polyps
**Sex**	1						
**Age**	-0.07	1					
**VAS scores**	0.192*	0.029	1				
**Lund Macky scores**	-0.101	0.092	0.294**	1			
**Frequency of variation**	-0.111	− 0.122*	-0.149	− 0.493**	1		
**Unilateral or bilateral**	-0.117	0.082	0.164*	0.471**	− 0.442**	1	
**Nasal polyps**	-0.025	-0.054	0.205**	0.425**	− 0.261**	0.214**	1

* means the correlation was significant (P < 0.05), at 0.05 grade (two-tailed test)

** means the correlation was significant (P < 0.05), at 0.01 grade (two-tailed test)

## Discussion

Anatomical variation is common in chronic sinusitis. But the role of anatomical variations in the pathogenesis of chronic sinusitis is controversial [14, 15]. Although many researchers have investigated the roles of anatomical variation in chronic sinusitis [8, 9, 14], detailed analyses of chronic sinusitis subgroups and elucidation of the relationship of anatomical variation and nasal polyps remain lacking.

Among the various anatomical variations included in this study, the incidence of agger nasi cells was highest, as reported by other researchers [16]. Various studies found that one anatomical variant of the paranasal sinuses could narrow the conduits of mucociliary clearance, predisposing the patient to rhinosinusitis [17]. Our study showed that anatomical variants associated with narrowing of the maxillary sinus ostium and surrounding structures (concha bullosa, uncinate process variations, paradoxical middle turbinate, and accessory ostia) are more common in CRS patients than controls. More interestingly, there was no significant difference in the frequency of anatomical variants between DCRS and controls. The frequency of anatomical variation in LCRS was higher than that in these two groups, so we speculated the pathogenesis of LCRS more likely be associated with anatomical variations.

Similarly, Jain [10] have suggested that anatomical abnormalities (particularly concha bullosa and accessory ostia) may play a role in the pathogenesis of LCRS but may not be very important in DCRS. In rhinosinusitis, extensive mucosal abnormalities may be the main cause of DCRS. However, some researchers believe that variations in the local anatomical structure do not underlie the pathogenesis of chronic sinusitis. For example, Tuli [18] and others have suggested that variations in the uncinate process do not affect the occurrence of chronic sinusitis.

Olfactory dysfunction is the main manifestation of the difference in VAS score between LCRS and DCRS. Previous study considered that olfactory dysfunction is a manifestation of an immense inflammatory response [19]. In addition, nasal polyposis is the independent risks factors for olfactory dysfunction in CRS [20], which is consistent with the correlation of polyps between LCRS and DCRS in our study.

At present, many clinicians use sinus CT as a basis for the diagnosis of chronic sinusitis and as an indicator of the severity of the disease. Ta [21] have reported that the overall correlation between the CT score and symptom score of CRS is poor. However, Devaraja [14] have reported that no relationship exists between the severity of symptoms and the severity on CT scans. Our study confirmed the existence of a correlation between symptom VAS score and Lund-Macky CT score, although the strength of this correlation was poor. This needs to be confirmed by further studies through surgery.

Karki [22] has reported no significant differences between nasal septum deviation and ipsilateral maxillary sinusitis. In addition, according to the statistics reported by authoritative researchers, nasal septum deviation is present in 20–31% of the general population, whereas the incidence of chronic sinusitis is only 10.9% [2]. Although some researchers believe that severe nasal septum deviation is a cause of chronic sinusitis, they also believe that the severity of nasal septum deviation is relevant, and the evaluation of nasal septum severity is affected by many factors. Therefore, nasal septum deviation was not included in this study. We believe that this aspect is a limitation of this study, and in future work, we plan to further evaluate the relationship between the severity of nasal septum deviation and the incidence of chronic sinusitis. Simultaneously, owing to the loss of clear outlines around anatomical structures in imaging, some of the data may have contained errors. In this study, we did not further compare anatomical variations of postoperative symptoms and CT score, and more effectively guide the choice of clinical surgery.

## Conclusion

**Fig. 2:**
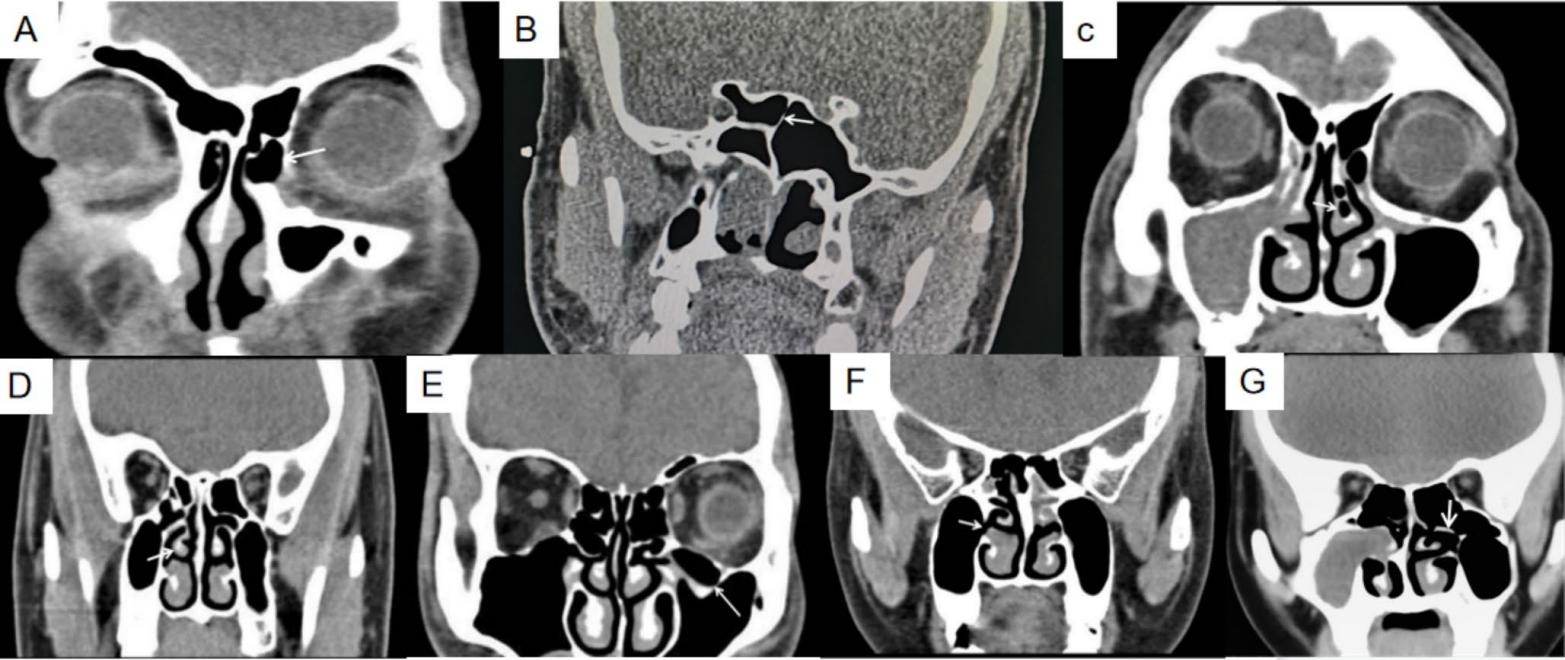
Different types of anatomical variation (coronal position): (A) Agger nasi cells. (B) Onodi cells. (C) Concha bullosa. (D) Paradoxical middle turbinate. (E) Haller cells. (F) Accessory ostia. (G) Uncinate process variations

We find that anatomical variants are more frequent in LCRS than DCRS and the pathogenesis of LCRS is possibly associated with anatomical variants. The frequency of anatomical variation is not associated with the occurrence of polyps. This would be addressed during surgery in the future (Fig. 2).
